# Postbiotics in rheumatoid arthritis: emerging mechanisms and intervention perspectives

**DOI:** 10.3389/fmicb.2023.1290015

**Published:** 2023-11-07

**Authors:** Zhen-Hua Ying, Cheng-Liang Mao, Wei Xie, Chen-Huan Yu

**Affiliations:** ^1^Zhejiang Key Laboratory of Arthritis Diagnosis and Research, Zhejiang Provincial People’s Hospital, Hangzhou Medical College, Hangzhou, China; ^2^Zhejiang University of Technology, Hangzhou, China; ^3^Key Laboratory of Experimental Animal and Safety Evaluation, Hangzhou Medical College, Hangzhou, China; ^4^Institute of Cancer and Basic Medicine, Chinese Academy of Sciences, Hangzhou, China; ^5^Cancer Hospital of the University of Chinese Academy of Sciences (Zhejiang Cancer Hospital), Hangzhou, China

**Keywords:** intestinal microbiota, metabolites, short chain fatty acids, Treg cells, Th17 cells

## Abstract

Rheumatoid arthritis (RA) is a prevalent chronic autoimmune disease that affects individuals of all age groups. Recently, the association between RA and the gut microbiome has led to the investigation of postbiotics as potential therapeutic strategies. Postbiotics refer to inactivated microbial cells, cellular components, or their metabolites that are specifically intended for the microbiota. Postbiotics not only profoundly influence the occurrence and development of RA, but they also mediate various inflammatory pathways, immune processes, and bone metabolism. Although they offer a variety of mechanisms and may even be superior to more conventional “biotics” such as probiotics and prebiotics, research on their efficacy and clinical significance in RA with disruptions to the intestinal microbiota remains limited. In this review, we provide an overview of the concept of postbiotics and summarize the current knowledge regarding postbiotics and their potential use in RA therapy. Postbiotics show potential as a viable adjunctive therapy option for RA.

## 1. Introduction

Rheumatoid arthritis (RA) is a long-term autoimmune disorder that causes persistent inflammation in the joints’ synovial membranes. It is often accompanied by subsequent destruction of the joint cartilage and erosion of bone (Alivernini et al., 2022). RA can develop at any age, with 80% of patients developing the disease between the ages of 35 and 50. Additionally, the number of female patients is two to three times higher than that of male patients (Sparks et al., 2023). Apart from impairing motor function, RA also has systemic effects on various organs including the respiratory, renal, and cardiac systems. Additionally, it can give rise to complications such as dry eye syndrome, pericarditis, anemia, and necrotizing vasculitis, significantly impacting patients’ everyday activities and professional life. Up to now, the pathogenesis of rheumatoid arthritis remains unclear. But most scholars believe that genetics and environmental factors may be the main triggers for RA (Desai et al., 2022; Guo et al., 2023; Lin et al., 2023).

Intestinal dysbiosis is one of the important causes of RA. Once the balance between beneficial bacteria, such as Bifidobacterium, and harmful bacteria, such as *Prevotella copri*, is disturbed, it can lead to an imbalanced ratio of intestinal microbiota and the proliferation of microorganisms related to RA. This imbalance can trigger inflammation and ultimately accelerate the progression of RA (Opoku et al., 2022; Romero-Figueroa et al., 2023). Probiotic intervention and fecal microbial transplantation (FMT) can restore the balance of the intestinal microbiota, improve intestinal dysbiosis, and prevent the occurrence and development of RA, and even achieve a cure. The connection with the gut microbiome has led to the use of oral probiotics or FMT as therapeutic strategies for RA (Wang et al., 2023; Zhao et al., 2023). However, as viable microorganisms, those strategies have some potential biosafety risks. Postbiotics are being used as novel food supplements and provide safer and higher quality products for controlling the microbial population compared to probiotics. Oral administration of postbiotics can enhance immunity, regulate intestinal microbiota, improve growth performance, and reduce the occurrence of diarrhea (Yeom et al., 2021; Jung et al., 2023). Especially, the metabolites derived from intestinal microbiota improved the intestinal barrier integrity and mediated the balance of Treg/Th17 cell ratio, resulting in the decreased release of serum IL-17 and expedite bone repair (Chen et al., 2021; Hanlon et al., 2022). Therefore, non-viable postbiotics have recently been considered as a better alternative. This paper summarizes the different types of postbiotics and their potential benefits in improving health and preventing diseases. It specifically focuses on the biological functions of postbiotics in preventing RA and highlights recent advancements in their clinical applications.

## 2. Roles of the gut dysbiosis on RA occurrence and development

**Figure 1:**
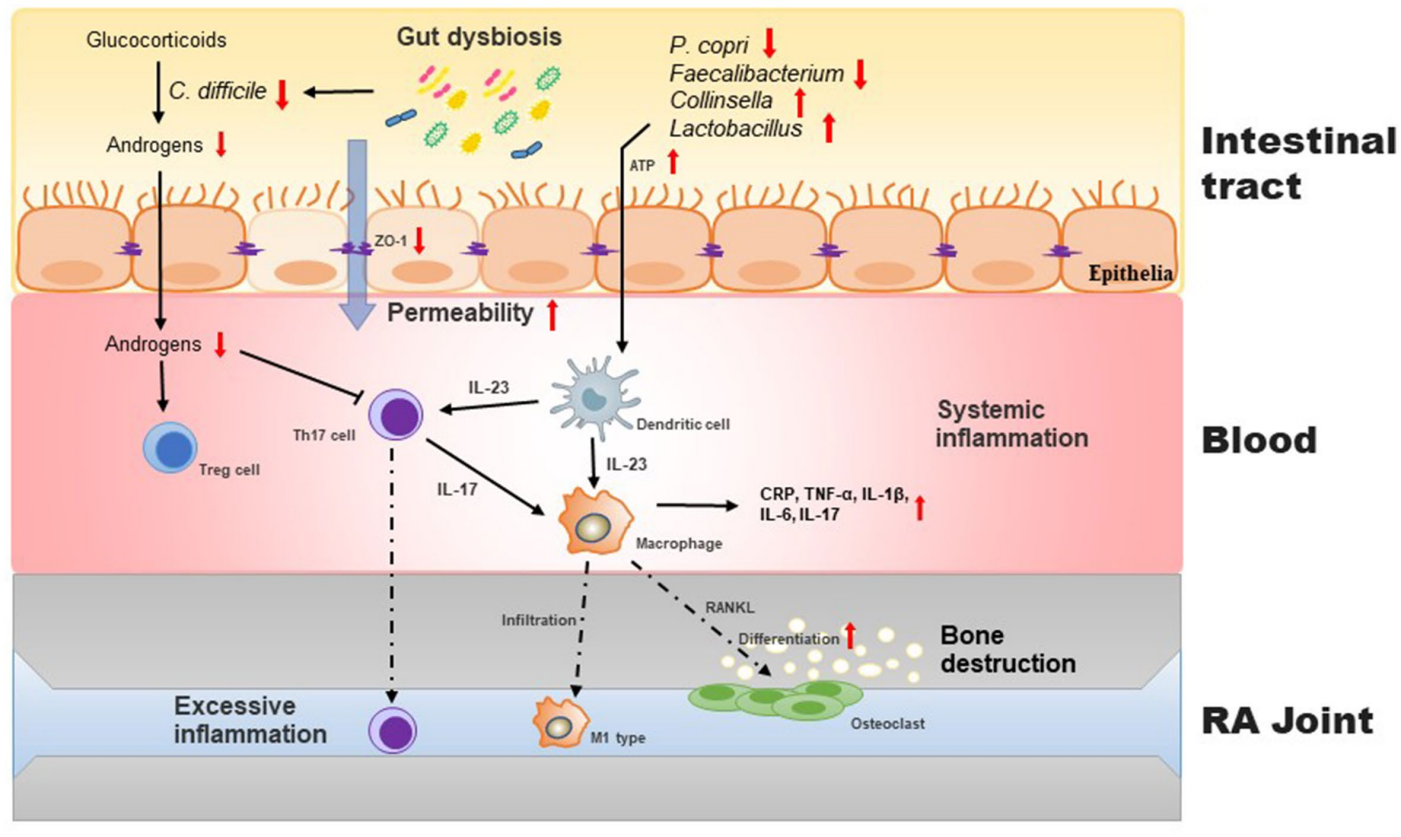
Intestinal barrier disrupted by gut dysbiosis facilitates induces imbalance of Th17/Treg cells and exacerbates inflammatory process in the pathogenesis of RA. During gut dysbiosis, the increased APT levels in gut stimulates the production of IL-23 in dendritic cells, which in turn promotes the differentiation of macrophage to osteoclast, resulting the bone destruction. Moreover, gut dysbiosis increases the intestinal permeability and subsequently aggravates host inflammatory responses. *C. difficile* regualtes the conversion of glucocorticoids to androgens that promote the proliferation of Treg cells.

The roles of intestinal microbiota in the occurrence of RA are mainly manifested in mucosal immunity and are associated with T cell differentiation, including regulatory T cells (Treg) and helper T (Th) cells. During intestinal dysbiosis, intestinal T cells undergo auto-activation, which can increase susceptibility to arthritis. Additionally, the metabolites produced by the intestinal microbiota can indirectly promote the development of RA (Figure 1).

### 2.1. Gut microbiota affects the Treg/Th17 balance

Gut microbiota is critical to affect the balance between Th17 cells and Treg cells. IL-17, which is secreted by Th17 cells, promotes the development of RA, while IL-10 and TGF-β1, which are secreted by Treg cells, control the progression of RA. Increasingly, studies have shown that the gut microbiota can influence the immune response to RA by regulating the homeostasis of Th17/Treg cells (Kotschenreuther et al., 2022; Dagar et al., 2023). In particular, the differentiation and expansion of Treg/Th17 cells are independently controlled by specific members of anaerobic bacteria. *P. copri* influences the ratio of Th1 to Th17 cells by mediating T helper cell differentiation, while *Bacteroides fragilis* enhances the anti-inflammatory effects of Treg cells through the expression of polysaccharide A, which interacts with TLR2 (Guerreiro et al., 2018; Alpizar-Rodriguez et al., 2019; Kitamura et al., 2021).

Compared to the germ-free mice, collagen-induced arthritis (CIA) mice showed a significant increase in serum IL-17 levels, as well as elevated levels of splenic CD8^+^ T cells and Th17 cells. Conversely, the levels of dendritic cells, B cells, and Treg cells were significantly reduced in CIA mice (Liu et al., 2016). Under germ-free conditions, the symptoms of the K/BxN mouse with autoimmune arthritis exhibited remarkable improvement, which was accompanied by notable reductions in serum autoantibody levels, splenic autoantibody-secreting cell, germinal centers, and splenic Th17 cell populations. Once a specific type of intestinal microbiota, such as segmented filamentous bacteria, is introduced, it becomes possible to reintroduce Th17 cells into the lamina propria of the small intestine. This, in turn, leads to the rapid production of antibodies and the development of arthritis (Wu et al., 2010). In addition, SKG mice harboring microbiota from patients with RA had increased numbers of Th17 cells in their intestines and developed severe arthritis when treated with yeast zymosan. T cells derived from naive SKG mice were co-cultured with *P. copri*-stimulated dendritic cells, thereby promoting the production of IL-17 in response to the arthritis-associated autoantigen RPL23A, leading to the rapid induction of arthritis (Maeda et al., 2016).

### 2.2. Gut microbiota affects the permeability of intestinal mucosa

The presence of intestinal dysbiosis can lead to an increase in the permeability of the intestinal mucosa, allowing conditionally pathogenic bacteria to translocate. This can result in heightened autoimmune inflammation and an increased risk of rheumatoid arthritis (Matei et al., 2021). The analysis of 16S ribosomal DNA sequencing data revealed a robust association between the prevalence of *Collinsella* and elevated concentrations of alpha-aminoadipic acid, asparagine, and IL-17A. More importantly, *Collinsella aerofaciens* enhances intestinal mucosal permeability and increases arthritis severity in HLA-DQ8 mice susceptible to collagen-induced arthritis. This effect is accomplished through the downregulation of tight junction proteins ZO-1 and occludin, as well as the upregulation of IL-17-mediated network cytokines, such as IL-1LA, CXCL1, CXCL5, and NF-κB1, in CACO-2 cells (Chen et al., 2016).

### 2.3. Gut microbiota affects the production of sex hormones

In the clinic, there are more women than men with RA, which may be attributed to the influence of intestinal microbiota on the regulation of sex hormones. Estradiol is dose-dependent and generally stimulates the production of pro-inflammatory factors, such as TNF-α and IL-1β, at low doses. When estrogen levels are elevated, such as during pregnancy, it can produce anti-inflammatory effects by inhibiting the signaling of pro-inflammatory factors, inducing the expression of anti-inflammatory factors (resulting in a Th2 phenotypic shift), and activating Tregs cells, respectively. Progesterone may reduce the severity of RA during pregnancy by promoting the production of Treg cells and inhibiting the differentiation of Th17 cells (Rizzetto et al., 2018; Brettle et al., 2022). Previous studies have demonstrated that *Clostridium difficile* encodes hydroxysteroid dehydrogenase and certain enzymes involved in the conversion of glucocorticoids to androgens. This can have an impact on the metabolism and activity of sex hormones, resulting in an immunomodulatory effect (Schmidt et al., 2020).

## 3. Application of postbiotics in RA treatment

**Figure 2:**
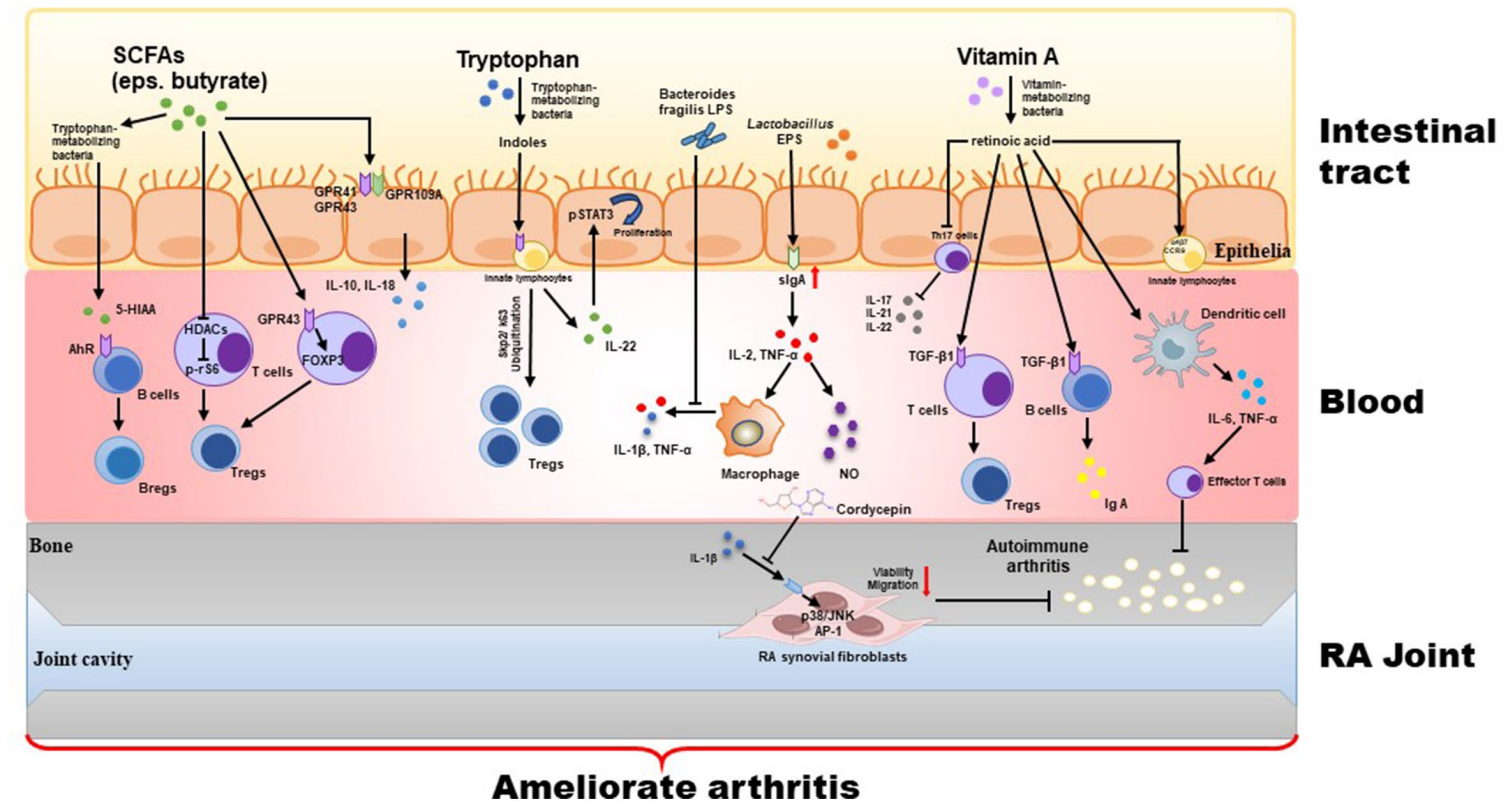
Treatment with postbiotics (including inactivated bacterial cells, cellular components, and their metabolites) strengthens intestinal tight junction via regulation of GPRs and HDACs signaling, and inhibits inflammatory response via mediating the Th17/Treg balance.

Probiotics refer to non-pathogenic microorganisms that are widely recognized for their ability to provide health benefits when consumed in adequate amounts by the host. As the scope and function of probiotics have expanded and deepened, researchers have discovered that not only can live bacteria perform probiotic functions, but some “non-live” bacterial components also exhibit significant health-promoting effects. These components include inactivated bacterial cells, components released by bacterial lysis after death, and bacterial metabolites. The bacterial components can include lipophosphatidic acid, cell surface proteins, and peptidoglycan, while metabolites include enzymes, peptides, short-chain fatty acids (SCFAs), and polysaccharides (e.g., extracellular polysaccharides). Depending on the source of the components, both the cell-free supernatant and the metabolites obtained from bacterial fermentation are usually referred to parabiotics. In contrast, the inactivated bacterial cells (lysed or unlysed), including cell wall fragments, bacteriophage cells, and cellular components, are referred to paraprobiotics. These inactivated bacteria and metabolites belong to the category of “postbiotics.” Although the mechanisms by which postbiotics are beneficial to human health have not yet been fully elucidated, it has been proven that postbiotics have various beneficial functions. These functions include protecting the epithelial barrier and possessing antitumor, antioxidant, and immunomodulatory properties. When consumed in sufficient quantities by the host, postbiotics can have a positive impact on microbiota homeostasis and/or host metabolic and signaling pathways, thereby influencing specific physiological, immunological, and metabolic functions (Figure 2).

Recently, postbiotic preparations can be obtained through various methods, including heat treatment, enzyme treatment, solvent extraction, exposure to γ or UV light, ultrasound, etc. In most cases, heat treatment is the common method used to inactivate probiotic organisms. Heat-inactivated probiotic cells, cell-free supernatants, and other active ingredients can produce beneficial effects, such as immunomodulation, balancing the intestinal microbiota, and regulating physiological functions. At the clinical level, products containing inactivated bacteria have been utilized to treat various gastrointestinal disorders, including bloating, diarrhea, and infantile colic. Inactivated bacteria have also proven to be useful in managing skin or respiratory allergies. Therefore, postbiotics have become a new means of improving intestinal microecology in the past 3 years.

### 3.1. Cell-free supernatant (CFS)

After centrifugation and filtration, CFS containing active metabolites can be obtained from microbial cultures. The supernatant of lactic acid bacteria usually includes low molecular weight compounds (e.g., hydrogen peroxide, organic acids, carbon dioxide, etc.) and high molecular weight compounds (such as bacteriocins, fine-like bacteriocins, and bacteriocin-like substances). *Lactobacillus acidophilus* and *Lactobacillus casei* supernatants have the ability to penetrate the intestinal mucosal barrier, decrease the secretion of TNF-α, and increase the production of anti-inflammatory IL-10, indicating its anti-inflammatory and antioxidant properties. *Bifidobacterium shorteri* supernatants can reduce the release of various inflammatory mediators in dendritic cells by activating the Toll-like receptor, thus safeguarding the immune system against pathogenic bacteria. Secreted protein HM0539 from *Lactobacillus rhamnosus* GG supernatant enhances intestinal mucin expression and prevents TNF-α-induced intestinal mucosal barrier damage.

### 3.2. SCFAs

SCFAs, the products of dietary fiber fermentation by intestinal microbiota, include acetic, propionic, and butyric acids, which are the most well-known components of postbiotics. Acetate and propionate are primarily used as substrates for mitochondrial oxidation in the liver and muscle, while butyrate is mainly utilized in the colon to provide energy to intestinal epithelial cells (Palmnäs-Bédard et al., 2022; Tan et al., 2023).

It is well known that autoimmune diseases, including RA, are often accompanied by intestinal barrier dysfunction. Zonulin is a peptide that regulates intestinal tight junctions, and is known to be closely linked with an impaired intestinal barrier, dysbiosis (an imbalance in the gut microbiota), and inflammation in both autoimmune mice and humans. Excitedly, the oral administration with zonulin antagonist larazotide acetate or butyrate, a specific intestinal microbiota metabolite, has been found to enhance intestinal barrier integrity. This restoration of the intestinal barrier has shown potential in reducing the onset of arthritis, indicating a protective effect of intestinal microbiota metabolites in the early stages of arthritis development (Tajik et al., 2020; Hecquet et al., 2023).

Compared to healthy controls, supplementation with microbial-derived butyrate reduces the severity of arthritis in mice. The levels of stool propionate and butyrate were significantly lower in RA patients and arthritic mice. Moreover, supplementation with butyrate has been observed to reduce the severity of RA through a Breg cell-dependent mechanism. This effect is achieved by increasing the levels of a serotonin-derived metabolite called 5-Hydroxyindole-3-acetic acid (5-HIAA), which in turn activates the aryl-hydrocarbon receptor (AhR) and then skews the B cell population. However, it was observed that mice deficient in B cells did not experience any benefits, highlighting the distinct role of B cells in the suppression of arthritis mediated by butyrate. Thus, butyrate could be used as a viable candidate for the treatment of systemic autoimmune diseases (Rosser et al., 2020). Furthermore, the regulation of butyrate mediated the differentiation of CD4^+^ T cells into Treg cells. These Treg cells can generate the anti-inflammatory cytokine IL-10, which in turn affects the function of Th17 cells and inhibits the expression of other inflammatory cytokines, and ultimately alleviated RA-related symptoms of the arthritic mice. However, it was also found that butyrate could not inhibit the expression of IFN-γ, although it could selectively inhibit IL-17A. Propionate ameliorated CIA by promoting the proliferation of Treg cells and elevating IL-10 levels both (Hui et al., 2019). Additionally, the consumption of a high-fiber diet enriched with resistant starch led to an increase in serum levels of intestinal acetate, propionate, and isobutyrate (Jiang et al., 2022).

In addition, alterations in bone metabolism are another significantly characteristic pathology in the progression of RA. Butyrate produced by *Firmicutes* bacteria, has been found to modulate the differentiation of Treg cells and promote bone formation in experimental animals. Clinical trial also demonstrated that purine metabolism is significantly reduced in RA patients when the intestinal microbiota is dominated by *P. copri*, indicating that the high abundance of *P. copri* in gut may affect the therapeutic efficacy of methotrexate to RA patients (Scher et al., 2013). Similarly, the abundant of *Prevotella copri* was closely related to the severity of the new-onset RA patients. The mice colonized with *P. copri* can increase the sensitivity to chemically induced colitis. However, there were not any significant changes observed in osteoblasts, suggesting the inhibition of SCFAs on bone loss of arthritic mice. Moreover, reduced bone erosion markers and improved Th1/Th17 ratios were also observed in individuals who followed a high-fiber diet for 4 weeks, leading to improved outcomes associated with RA. This intervention induces up-regulation of insulin-like growth factor 1 (IGF-1), which stimulates osteoblast proliferation and bone remodeling (Häger et al., 2019). Additionally, SCFAs produced by microbial catabolism have been shown to have the same effects in mice (Dürholz et al., 2020; Rosser et al., 2020).

Mechanically, SCFAs can activate immunity-related pathways by binding to G protein-coupled receptors (GPCRs) including GPR41, GPR43 and GPR109A. Acetic, propionic, and butyric acids primarily activate GPR41 and GPR43, while butyric acid also activates GPR109A (D'Souza et al., 2017). Butyrate can bind to GPR109A on the intestinal epithelial cells (IEC), macrophages, and dendritic cells, causing the secretion of IL-10. This, in turn, induces the differentiation of Treg cells. SCFAs can stimulate inflammation by binding to GPR43 on the vesicles of IEC, thereby activating downstream of IL-18, which is involved in repairing and maintaining the integrity of the intestinal barrier (Singh et al., 2014; Macia et al., 2015). Furthoremore, SCFAs can enhance the ability of intestinal T cells to express the transcription factor Foxp3 by activating GPR43 on T cells, thereby regulating the development and differentiation of Treg cells (Balakrishnan et al., 2021; Duan et al., 2023). Notably, SCFAs also can act as the inhibitors of HDACs and subsequently accelerate the differentiation and function of T cells to regulate the immune tolerance of the body. Studies have shown that butyric acid, as a violent inhibitor of HDACs, can increase the acetylation of p70S6K and phosphorylation of rS6, thereby expediting T-cell differentiation into effector and regulatory cells via activation of mTOR-S6K pathway (Park et al., 2015). It has been shown than SCFAs can promote IL-22 production by splenic CD4^+^ T cells and innate lymphocytes in wild-type C57BL/6 J mice through the inhibition of HDACs and binding to GPR41 (Li et al., 2018; Yang et al., 2020). Notably, those inhibitory effects of SCFAs on HDAC activity is not limited to immune cells in the intestine, but also extends to other tissues and organs through the circulation. The inhibition of HDACs leads to the inactivation of NF-κB, which subsequently reduces the release of inflammatory cytokines in RA patients and arthritic mice (Kim et al., 2018; Mao et al., 2023; Vijaykrishnaraj et al., 2023).

### 3.3. Exopolysaccharides (EPS)

Microorganisms can secrete extracellular polysaccharides (also called as exopolysaccharides) to the outside of their cells during growth. Some EPS can act as virulence factors for pathogens or help pathogens to adhere and colonize the gut; on the other hand, EPS can also produce beneficial effects on the host (Liu et al., 2019; Dahiya and Nigam, 2022). In addition, EPS have antioxidant, anti-infective and anticancer effects (Kawanabe-Matsuda et al., 2022; Sharma et al., 2023; Srinivash et al., 2023; Zeinivand et al., 2023).

As previously reported, EPS isolated from *Lactobacillus* in yogurt can stimulate NK cell activation and regulate immune response. It also increased phagocytosis of macrophages, stimulated NO secretion, and resisted immunosuppression in cyclophosphamide-exposed mice by promoting the production of sIgA, IL-2 and TNF-α in the intestinal mucosae (Makino et al., 2016). Ebosin, a novel EPS isolated from *Lactobacillus rhamnosus* remarkably inhibited IL-1β-mediated MAPK and NF-κB pathways in rat fibroblast-like synoviocytes and then reduced arthritogenic autoantibodies in collagen+lipopolysaccharide (LPS)-induced arthritic mice (Nowak et al., 2012; Zhang et al., 2016, 2022).

### 3.4. Tryptophan metabolites

Tryptophan is an essential amino acid that cannot be synthesized by the animal body itself. Food is the primary source of intake. Most intestinal microbiota in animals, except for viruses and archaea, can metabolize tryptophan through various pathways to produce a range of biologically active molecules, including indole, tryptamine, indole ethanol, indole propionic acid, indole lactic acid, indole acetic acid, and indole acrylic acid which are primarily produced by specific strains as summarized in Supplementary Table S1.

AhR is a sensor of microbial metabolites that plays a role in the development of innate lymphocytes and intraepithelial lymphocytes, thereby exerting antimicrobial and anti-inflammatory effects (Stone and Williams, 2023). Many microbial tryptophan metabolites could activate the AhR. Indole, 2-indolone, indoleacetic acid, and kynurenine can activate the AhR, thereby mediating a variety of immune responses (Li, 2023). Indole-3-aldehyde, a metabolite of *Lactobacillus Royce* D8, activates the AhR, promotes the secretion of IL-22 by innate lymphocytes, and upregulates the expression of p-STAT3 in IEs that in turn promotes the proliferation of intestinal epithelial cells and facilitates the repair of damaged intestinal mucosa. Activation of AhR by tryptophan metabolites in the intestine enhances the function of group 3 innate lymphoid cells (Hou et al., 2018), and promotes Treg cell generation via the Skp2/K63-ubiquitination pathway to prevent and treat RA, indicating the potential of AhR agonists and tryptophan metabolites (Zhang et al., 2023).

### 3.5. Vitamins and their metabolites

In addition to the vitamins obtained from foods, animals can also metabolize and synthesize some vitamins (mainly vitamin K2 and B family) by intestinal microbiota as summarized in Table 1 (Yoshii et al., 2019). It is known that vitamin B1 can be synthesized by *Bifidobacterium fragilis*, *Prevotella*, and *Bifidobacterium*, vitamin B2 by *Lactobacillus plantarum*, *Lactobacillus fermentum*, and *Lactobacillus fragilis*, vitamin B9 by *Bifidobacterium bifidum* and *Bifidobacterium longum*, while vitamin B12 by *Propionibacterium feldsponenum*, *Salmonella*, and *Lactobacillus roehlis* (Barone et al., 2022). Vitamin K2 is endogenously synthesized by intestinal bacteria, such as *Viridans Streptococci*, *B. subtilis natto*, *L. lactis*, *L. reuteri*, *Pichia pastoris*, and *Flavobacterium* sp.

**Table 1:** The origin and function of vitamin B family and vitamin K.

Vitamins	Metabolizing bacteria	Function	References
Vitamin B1	*Bifidobacterium fragilis*	Promoting immune defense of the intestinal mucosa; Promoting macrophage proliferation and B cell differentiation	Kunisawa et al. (2015), Sabui et al. (2022), and Stambuk et al. (2009)
	*Prevotella copri*		
	*Clostridium difficile*		
	*Lactobacillus casei*		
	*L. curvatus*, *L. plantarum*		
	*Ruminococcus lactaris*		
	*B. bifidum*, *B. infantis*		
	*Fusobacterium varium*		
Vitamin B2 and its intermediate 6-hydroxy methyl-8-d-ribityllumazine	*L. plantarum*, *L. fermentum*	Promoting differentiation of naïve B cells; Inducing IL-17 and IFN-γ production by MAIT cells	Vogl et al. (2007) and Birkenmeier et al., 2015
	*B. fragilis*		
	*P. copri*		
	*C. difficile*		
	*R. lactaris*		
Vitamin B6	*B. fragilis*, *B. longum*	Maintaining Th1/Th2 balance	Lee et al. (2023) and Wan et al. (2022)
	*P. copri*		
	*Collinsella aerofaciens*		
	*Helicobacter pylori*		
Vitamin B9 and its metabolite 6-FP	*B. fragilis*	Promoting Treg cell differentiation via activating FR4; Inhibition of MAIT cells	Kunisawa et al. (2012) and Wan et al. (2022)
	*P. copri*		
	*C. difficile*		
	*L. plantarum*, *L. delbrueckii*, *L. reuteri*		
	*Streptococcus thermophilus*		
	*Fusobacterium varium*		
	*S. enterica*		
Vitamin B12	*Bacteroides fragilis*	Activation of CD8^+^ cells and NK-T cells	Barone et al. (2022), Degnan et al. (2014) and Tanaka et al. (2017)
	*P. copri*		
	*C. difficile*		
	*Faecalibacterium prausnitzii*		
	*R. lactaris*		
	*L. plantarum*, *L. cortniformis*, *L. reuteri*		
	*B. animalis*, *B. infantis*, *B. longum*		
	*F. varium*		
Vitamin K2	*Viridans Streptococci*	Inhibit osteoclastogenesis via Osteocalcin, Gla, nuclear steroid and xenobiotic receptor	Yuan et al. (2020) and Mladěnka et al. (2022)
	*B. subtilis natto*		
	*L. lactis*, *L. reuteri*		
	*Pichia pastoris*		
	*Flavobacterium* sp.		

After being absorbed and metabolized by the body, retinoic acid, the main metabolites of vitamin A, promotes the production of Treg cells by CD4^+^ T cells and immunoglobulin A by B cells via activation of TGF-β1 pathway (Qiu et al., 2023; Shri Preethi et al., 2023). A deficiency in retinoic acid inhibits the differentiation of Th17 cells in the lamina propria of the small intestine in mice and reduces the secretion of IL-17, IL-21, and IL-22. In addition, retinoic acid induces innate lymphocytes to express α4β7 and CCR9 molecules, which helps these cells home to the intestine and promotes a balance of Th17/Treg cells in the intestinal-associated lymphoid tissue (Manhas et al., 2022). When the body is in an inflammatory state, retinoic acid can induce dendritic cells to produce the pro-inflammatory cytokines IL-6 and TNF-α. This, in turn, promotes the differentiation of effector T cells and helps protect the intestinal mucosal barrier function against collagen-induced autoimmune arthritis (McBride et al., 2023).

### 3.6. Others

It has been reported that RA patients had remarkably lower levels of gut *Bacteroides* species. Treatment with *Bacteroides fragilis* LPS significantly suppressed TNF-α and IL-1β production in *Escherichia coli* LPS-exposed macrophages and improved the development of CIA in mice (Kitamura et al., 2021).

Cordycepin (3′-deoxyadenosine), one of the major bioactive compounds in *Cordyceps militaris* and fermented *Hirsutella sinensis* mycelium extract, has been demonstrated to exert anticancer and anti-inflammatory activities as well as immunomodulation (Alhakamy and Fahmy, 2022; Liu et al., 2022; Cheng et al., 2023; Priya et al., 2023). Cordycepin significantly inhibited the viability and migration of RA synovial fibroblasts via blocking IL-1β-induced p38/JNK and AP-1 activation, thereby preventing inflammation of RA (Noh et al., 2009).

## 4. Dosage and safety in use

Due to the complex composition and significant variations in composition produced by different strains, there are challenges in standardizing the dosage of postbiotics in current production practices, as well as in relevant formulation standards. Although national and international standards on the recommended intake of probiotics are relatively mature, no standards have been issued yet to regulate or recommend the intake of the postbiotics.

According to the clinical trials, studies focusing on parabiotic bacteria have shown that the number of dead bacteria corresponds to the number of live bacteria. However, due to the variability of metabolites, studies focusing on postbiotic metabolites have not yet determined specific dosages. Whether the dosage of postbiotics can be recommended based on the dosage of probiotics during practical application requires further observation.

**Figure 3:**
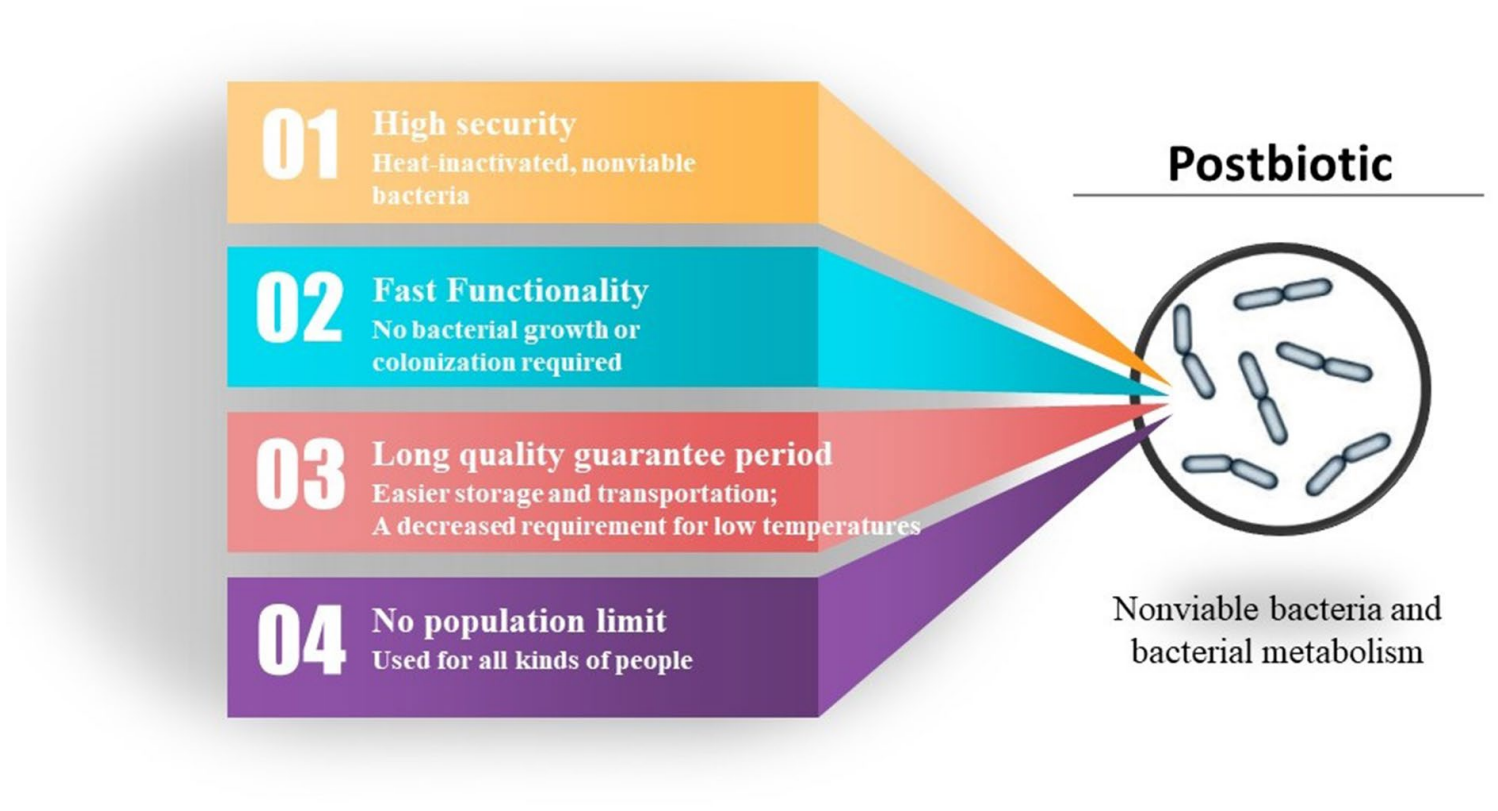
Advantages of postbiotic.

From an economic standpoint, the advantages of parabiotics over probiotics include an extended shelf life, easier storage and transportation, and a decreased requirement for low temperatures. The possibility of utilizing a repetitive production process and implementing more precise dosing controls are additional advantages of postbiotics in comparison to probiotics. The undeniable advantage of postbiotics is that it circumvents the issue of the organism potentially acquiring antibiotic resistance genes and virulence factors, which can accumulate in the body when using probiotics. Postbiotics eliminate the need for exposure to live microorganisms, which is particularly crucial for children with underdeveloped immune systems and compromised gut barriers. Compared to paraprobiotics, the composition of parabiotic components is easier to determine, the dosage and efficacy are more stable, and there is relatively less variation among different probiotic sources (Figure 3).

## 5. Future challenges and prospects

Postbiotics, derived from intestinal microorganisms, can serve various functions in the intestinal tract and throughout the body to treat and prevent a variety of autoimmune diseases. Recently, there has been a growing focus on studying the effects of probiotics and their derivatives on bone metabolism and function. Many studies have shown the potential of postbiotics in the treatment of osteoarthritis and RA. Therefore, the current clinical reliance on antirheumatic drugs to manage the condition of RA could be changed by establishing an evidence-based link between RA and the intestinal microbiome. The clinical application of the new strategy “targeting intestinal microbiota” can not only alleviate the symptoms of RA, but also offer personalized treatment and reduce the economic burden on patients and society.

As a burgeoning field, there are still numerous challenges that need to be addressed. Firstly, the definition and scope of postbiotics still need to be refined and expanded. The specific bacterium-derived small RNA, polypeptides, and exosome-like nanoparticles also exert noticeable bioactivities. Secondly, most of the products are still in the experimental stage or are used as food supplements. The specific mechanism of postbiotics against arthritis induced by various genetic or environmental factors has not been fully elucidated. Only after clarifying the characteristic active ingredients that contribute to the effectiveness of postbiotics can qualitative and quantitative analytical methods be developed to control the quality of postbiotics. Finally, SCFAs can be obtained by fermenting plant polysaccharides with intestinal microorganisms. CFS are obtained through centrifugal filtration, and bacterial lysates are prepared through chemical or mechanical degradation. Therefore, the production of different postbiotics involves various methods, which makes it challenging to establish standardized criteria for their industrial production. However, in any case, the potential of postbiotics in autoimmune diseases and other fields still deserves attention and anticipation.

## Author contributions

Z-HY: Funding acquisition, Project administration, Writing – original draft. C-LM: Conceptualization, Writing – original draft. WX: Visualization, Writing – review & editing. C-HY: Funding acquisition, Project administration, Supervision, Writing – review & editing.
