# Supplementary material for: Postbiotics in rheumatoid arthritis: emerging mechanisms and intervention perspectives
Source: Front Microbiol. 2023 Nov 7;14:1290015. doi: 10.3389/fmicb.2023.1290015 (PMC10662086; doi:10.3389/fmicb.2023.1290015)
Supplement: Supplementary file 1 [file Table_1.docx]

Table S1 The origins of tryptophan metabolites

| Tryptophan metabolites | Metabolizing bacteria | References |
| --- | --- | --- |
| Indole | *Bacteroides thetaiotaomicron*, *Bacteroides ovatus* | Devlin et al., 2016;  Roager and Licht, 2018 |
|  | *Clostridium bifermentans*, *Clostridium tetani*, *Clostridium sordellii* |  |
|  | *Enterococcus spp.* |  |
| 3-methylindole | *Bacteroides thetaiotaomicron*, *Butyrivibrio fibrisolvens* | Roager and Licht, 2018; Russell et al., 2013 |
|  | *Clostridium barati*, *Clostridium sporogenes* |  |
|  | *Lactobacillus* |  |
|  | *Eubacterium rectum* |  |
| Indolyl-3-propionic acid | *Clostridium botulinum*, *Clostridium thermostable*, *Clostridium perfringens*, *Clostridium sporogenes* | Dodd et al., 2017;  Williams et al., 2014 |
|  | *Peptostreptococcus saccharolyticus*, *Peptostreptococcus anaerobius* |  |
| Indole-3-lactic acid | *Bacteroides thetaiotaomicron*, *Bacteroides eggethii*, *Bacteroides ovale*, *Bacteroides fragilis* | Dodd et al., 2017;  Russell et al., 2013 |
|  | *Bifidobacterium adolescentis*, *Bifidobacterium bifurcatum* |  |
|  | *Clostridium barati*, *Clostridium perfringens*, *Clostridium sporogenes* |  |
|  | *Escherichia coli*, *Eubacterium rectum*, *Eubacterium cylindricum* |  |
|  | *Lactobacillus muris*, *Lactobacillus reuteri* |  |
| Indole-3-acetic acid | *Bacteroides thetaiotaomicron*, *Bacteroides eggerthii*, *Bacteroides fragilis* | Roager and Licht, 2018; Russell et al., 2013 |
|  | *Clostridium barati*, *Clostridium difficile*, *Clostridium paraputrificum* |  |
|  | *Eubacterium hallii*, *Eubacterium cylindricum* |  |
